# A Novel Series of Acylhydrazones as Potential Anti-*Candida* Agents: Design, Synthesis, Biological Evaluation and In Silico Studies

**DOI:** 10.3390/molecules24010184

**Published:** 2019-01-06

**Authors:** Anca-Maria Borcea, Gabriel Marc, Ioana Ionuț, Dan C. Vodnar, Laurian Vlase, Felicia Gligor, Andreea Pricopie, Adrian Pîrnău, Brîndușa Tiperciuc, Ovidiu Oniga

**Affiliations:** 1 Department of Pharmaceutical Chemistry, “Iuliu Haţieganu” University of Medicine and Pharmacy, 41 Victor Babeş Street, 400012 Cluj-Napoca, Romania; borcea.anca@umfcluj.ro (A.-M.B.); pricopie.andreea@umfcluj.ro (A.P.); brandu32@yahoo.com (B.T.); onigao65@yahoo.com (O.O.); 2 Preclinic Department, Pharmacy Specialization, Faculty of Medicine, Lucian Blaga University of Sibiu, 2A Lucian Blaga Street, 550169 Sibiu, Romania; felicia.gligor@ulbsibiu.ro; 3 Department of Food Science and Technology, University of Agricultural Sciences and Veterinary Medicine, 3-5 Mănăştur Street, 400372 Cluj-Napoca, Romania; dan.vodnar@usamvcluj.ro; 4 Department of Pharmaceutical Technology and Biopharmaceutics, “Iuliu Haţieganu” University of Medicine and Pharmacy, 41 Victor Babeş Street, 400012 Cluj-Napoca, Romania; laurian.vlase@umfcluj.ro; 5 National Institute for Research and Development of Isotopic and Molecular Technologies, 67-103 Donath Street, 400293 Cluj-Napoca, Romania; adrian.pirnau@itim-cj.ro

**Keywords:** thiazole, anti-*Candida*, acylhydrazone, lanosterol 14α-demethylase, molecular modeling, ADMET

## Abstract

In the context of an increased incidence of invasive fungal diseases, there is an imperative need of new antifungal drugs with improved activity and safety profiles. A novel series of acylhydrazones bearing a 1,4-phenylene-bisthiazole scaffold was designed based on an analysis of structures known to possess anti-*Candida* activity obtained from a literature review. Nine final compounds were synthesized and evaluated in vitro for their inhibitory activity against various strains of *Candida* spp. The anti-*Candida* activity assay revealed that some of the new compounds are as active as fluconazole against most of the tested strains. A molecular docking study was conducted in order to evaluate the binding poses towards lanosterol 14α-demethylase. An in silico ADMET analysis showed that the compounds possess drug-like properties and represent a biologically active framework that should be further optimized as potential hits.

## 1. Introduction

The continuous rise of fungal infections due to the increased number of immunocompromised patients is currently a major concern. Different *Candida* strains, including *C. albicans*, *C. krusei* and *C. parapsilosis* are known to be etiological agents of human infections. *C. albicans* is the most prevalent cause of invasive fungal diseases, being a serious public health problem due to high rates of mortality [1]. Although *C. albicans* accounted for more than half of the cases of candidemia in European countries, a progressive increase of non-albicans infections has been reported. The incidence rates for candidemia are 16.3% for *C. parapsilosis* and 2–5% for *C. krusei* [2,3]. The occurrence of *C. parapsilosis* as a nosocomial pathogen continues to rise, this non-albicans *Candida* strain being commonly associated with prosthetic surfaces, parenteral nutrition and central venous catheters [4].

The mechanism of action of azole antifungals involves the blockage of ergosterol biosynthesis through inhibition of lanosterol 14α-demethylase (CYP51), a member of the cytochrome P450-dependent superfamily. These antifungal agents bind in a strong and reversible manner to the heme iron atom situated in the catalytic site of the enzyme. As a result of CYP51 inhibition, the normal function of fungal membrane is affected due to reduced rigidity and increased permeability and fluidity [5]. Development of different mutations on CYP51 has led to a decreased binding affinity of the drug to the target enzyme and to an increased frequency of drug resistance [6]. The emerging fungal resistance constitutes a widespread problem for the treatment of invasive infections caused by *Candida* species.

The important role of CYP51 in fungal growth explains the actual interest in maintaining this enzyme as a target for the development of new anti-*Candida* drugs, but, in the same time, efforts are being made in order to discover compounds with a different inhibitory mechanism.

To accelerate the progress of development of more effective antifungal drugs, the identification of hit or lead compounds needs to be done. Recently published studies revealed that substituted thiazoles exhibit anti-*Candida* activity [7,8,9]. Furthermore, hydrazone and acylhydrazone derivatives, which possess an azomethine group −N=CH−, have been developed and evaluated for their inhibitory activity on different *Candida* spp. [10,11,12,13,14].

Gidaro et al. recently developed a ligand-based pharmacophore hypothesis, based on structurally similar molecules with confirmed in vitro anti-*Candida* activity. In their study, the importance of several chemical features of the theoretical pharmacophore hypothesis was observed: a central feature represented by a heterocycle (aromatic ring and hydrophobic feature—Ar-HF) containing a hydrogen bond acceptor—HBA (thiazole containing the nitrogen atom) linked to a hydrogen bond donor site—HBD and a second HBA site (hydrazone and carbonylic moiety) and to other aromatic or hydrophobic features (phenyl) [15].

**Figure 1:**
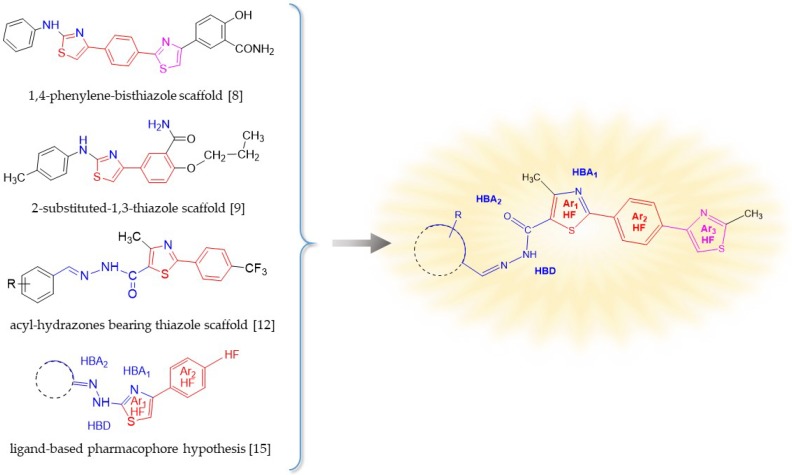
The design of the new 1,4-phenylene-bisthiazole acylhydrazones.

Based on this ligand-based pharmacophore hypothesis and on several molecules with in vitro anti-*Candida* activity, herein we focused on designing a new scaffold that contains some of the above-mentioned chemical features, among others, that are found in different active compounds, as illustrated in Figure 1. Association of various structural moieties, which have proved to possess an important antifungal effect, on the same chemical structure represents an important strategy for the development of new drugs, leading to compounds with improved biological activity.

Following that, nine new 1,4-phenylene-bisthiazole acylhydrazones were synthesized and evaluated in vitro for their inhibitory activity against different *Candida* strains. Moreover, computational approaches in drug discovery domain, including molecular docking and prediction of ADMET properties, are of great importance because they could reduce the number of experiments required, which are money and time-consuming processes [16]. The pharmacological activity of compounds is closely related to their pharmacokinetics and pharmacotoxicology. Among the interaction of a drug with its molecular target, properties such as absorption, distribution, metabolism, excretion and toxicity (ADMET) influence its efficacy and safety. In order to predict their binding poses towards CYP51 and to evaluate the drug-like properties of the synthesized compounds, a molecular docking study and an in silico ADMET prediction were also carried out.

## 2. Results and Discussion

### 2.1. Chemistry

**Scheme 1:**
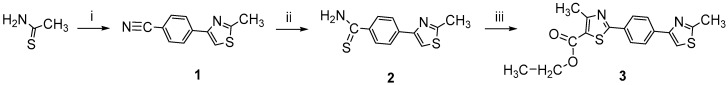
The synthesis of the 1,4-phenylene-bisthiazole carboxylate **3**: (i) 4-(2-bromoacetyl)- benzonitrile/EtOH, reflux; (ii) hydrogen sulfide gas, triethylamine, room temperature; (iii) ethyl 2-chloroacetoacetate/EtOH, reflux.

Ethyl 4-methyl-2-(4-(2-methylthiazol-4-yl)phenyl)thiazole-5-carboxylate (**3**) was previously synthesized and characterized by our research group [17]. The reaction sequence used for its synthesis is summarized in Scheme 1.

Briefly, the synthetic protocol is based on the Hantzsch condensation of thioacetamide with 4-(2-bromoacetyl)benzonitrile, followed by the treatment of 4-(2-methylthiazol-4-yl)benzonitrile **1**, with hydrogen sulfide gas, in the presence of triethylamine. Then, ethyl 4-methyl-2-(4-(2-methylthiazol-4-yl)phenyl)thiazole-5-carboxylate **3** was obtained by reacting the thioamide derivative **2** with ethyl 2-chloroacetoacetate [17].

**Scheme 2:**
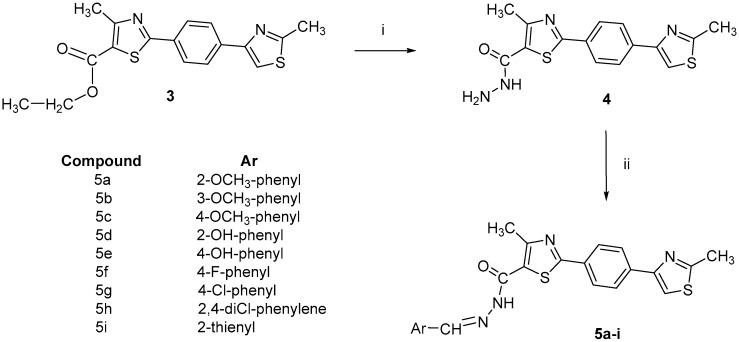
The synthesis of the target 1,4-phenylene-bisthiazole acylhydrazones **5a**–**i** (i) NH_2_-NH_2_/EtOH, reflux; (ii) Ar-CHO/H_2_SO_4_/EtOH, reflux.

The target compounds, 1,4-phenylene-bisthiazole acyl-hydrazones **5a**–**i**, were obtained according to the route illustrated in Scheme 2.

The hydrazide derivative **4** was subsequently synthesized by refluxing ethyl 4-methyl-2-(4-(2-methylthiazol-4-yl)phenyl)thiazole-5-carboxylate (**3**) with hydrazine hydrate in absolute ethanol (yield 69%). The final acylhydrazone derivatives **5a**–**i** were obtained in good yields (73–89%), by reacting the hydrazide **4** with various aromatic or heteroaromatic aldehydes in absolute ethanol.

All target compounds and their key intermediate **4** were successfully characterized by elemental analysis and FT-IR, ESI-MS, ^1^H- and ^13^C-NMR spectral data. The results of the C, H, N, S quantitative elemental analysis of the obtained compounds were within ±0.4% of the theoretical values.

Analyzing the IR spectra of the newly synthesized acylhydrazones, we could identify specific signals such as: a sharp signal with medium intensity at 3112–3094 cm^−1^, corresponding of the stretching vibration of the C_5_–H from the thiazole ring, a strong signal of high intensity at 1682–1658 cm^−1^ due to the presence of the C=O bond, an absorption band at 3281–3241 cm^−1^ due to the stretching vibration of the N–H bond, as well as a characteristic band at 1611–1598 cm^−1^, which was attributed to the stretching of the C=N double bond.

Signals that are specific for each final compound could also be observed: two strong signals found between 1265–1252 cm^−1^ and 1039–1025 cm^−1^, corresponding to the asymmetric and symmetric stretching vibration of the C–O–C ether bond in compounds **5a**–**c**, a wide absorption band characteristic for the stretching vibration of the O–H bond at 3419 cm^−1^ for compound **5d** and at 3418 cm^−1^ for compound **5e**, a characteristic signal at 1231 cm^−1^ due to the C–F bond in compound **5f** and strong signals between 743–701 cm^−1^ due to the C–Cl vibrations in compounds **5g** and **5h**.

The mass spectra recorded for all newly synthesized compounds revealed the presence of the molecular ion peaks (M + 1), as expected from their molecular formulas.

In the ^1^H-NMR spectra of the key intermediate **4**, we can observe two broad signals due to the NH and NH_2_ protons at 9.61 ppm and 4.63 ppm, respectively. The absence of NH_2_ protons signal, the shift of the signal corresponding to the NH proton from 9.61 ppm, in compound **4**, to 11.75–11.93 ppm, in compounds **5a**–**i**, and the presence of the N=CH proton and additional aromatic protons, confirmed the condensation between the carbohydrazide **4** and the corresponding aromatic aldehydes. In the ^13^C-NMR spectra of compounds **5a**–**i**, the presence of the C=N peaks from the azomethine group at 139.3–149.0 ppm proved once again that the synthesis of the acylhydrazones has been successfully accomplished.

### 2.2. Anti-Candida Activity Assay

The newly synthesized compounds were evaluated for their in vitro anti-*Candida* inhibitory activity, using four human opportunistic pathogenic *Candida* strains. The obtained results were compared with those of fluconazole, used as reference drug, and are illustrated in Table 1 and Table 2.

The best inhibitory activity on both *Candida albicans* strains was obtained when the Ar substituent was a substituted phenyl ring, in *orto* position with a hydroxyl group (compound **5d**) or in *para* position with a fluoro atom (compound **5f**). Both compounds presented the same anti-*Candida* activity as fluconazole on *C. albicans* strains (MIC = 15.62 µg/mL, MFC = 31.25 µg/mL). Compounds **5d**, **5f**, **5g** and **5h** were the most active in our series on *Candida krusei*, showing MIC and MFC values equal to those of fluconazole (MIC = 15.62 µg/mL, MFC = 31.25 µg/mL). The *Candida parapsilosis* strain seemed to be less susceptible to almost all our tested compounds, compound **5g** being the only one with an inhibitory activity equal to the one of our reference drug (MIC = 7.81 µg/mL, MFC = 15.62 µg/mL).

Some structure-activity relationships (SAR) could be observed based on the obtained MIC and MFC values. Compound **5d** (2-OH-phenyl derivative) showed a 2-fold greater anti-*Candida* activity, when compared with compound **5e** (4-OH-phenyl derivative), suggesting that the presence of the hydroxyl group in *orto* position of the phenyl ring increases the anti-*Candida* activity.

When Ar substituent group was represented by 4-F-phenyl or 4-Cl-phenyl, the corresponding target compounds **5f** and **5g** showed superior anti-*Candida* activity compared to compounds substituted with 4-OH-phenyl or 4-OCH_3_-phenyl. Therefore, *para*-substitution of the phenyl ring with a halogen atom could enhance the anti-*Candida* activity, fact which was also observed by Secci et al. [18] and by our group in our previous studies [12,19,20]. When Ar substituent group was 2,4-di-Cl-phenylene (compound **5h**), a decrease in the inhibitory activity on *Candida parapsilosis* was observed. No improvement in the anti-*Candida* activity was observed when the phenyl nucleus from the Ar substituent group was replaced by the thienyl moiety (compound **5i**).

Antifungal agents are considered to have fungicidal activity if the MFC/MIC ratio is ≤4 and fungistatic effect if the MFC/MIC ratio is >4. All our tested compounds have a MFC/MIC ratio equal to 2, indicating that these compounds behave as fungicidal agents [21].

### 2.3. Molecular Docking

In the last few years, lanosterol 14α-demethylase has been intensively studied for the discovery and development of novel antifungal agents, which are capable of inhibiting it [22,23]. The present study is focused on analyzing the binding affinity of our tested compounds **5a**–**i** towards both the active site and the access channel of the enzyme. The predicted best binding conformation of each compound to the catalytic site of lanosterol 14α-demethylase, in terms of binding energy, expressed as the variation of Gibbs free energy (ΔG), as well as the calculated inhibition constant (Ki) and the statistical analysis of the cluster containing the top binding conformation are presented in Table 3.

All tested compounds **5a**–**i** have good to very good binding affinity to lanosterol 14α-demethylase of *Candida albicans*, their binding energy values being under −11 kcal/mol. Statistically speaking, for many compounds in our series and especially for those with the best binding affinity to the target enzyme, it could be observed that most of the resulted conformations are in the same 2Å cluster with the best binding conformation.

A detailed energy analysis of the cluster of the top binding conformation provided that the average value of the Gibbs free energy is high and close to the best binding conformation found, fact that is well correlated with a low standard deviation of the values around the average value. A low standard deviation of the root-mean-square deviation of the three-dimensional Cartesian coordinates of each conformation from the top binding cluster found, led us to conclude that each individual pose is relatively close to others found in the same cluster. Moreover, the presence of a reduced number of clusters with residual poses, that are not found in the top cluster, is correlated with the idea that the high number of conformations found for each compound, in the top cluster, relates to the constant binding of the compounds in the same zone of the protein, interacting with the same amino acid residues. Thus, we can conclude that this in silico assay has good reproducibility.

In order to evaluate how different substituents, located on the Ar group, influence the binding affinity of the tested compounds, we have visually analyzed and compared the binding mode of the compounds to the target lanosterol 14α-demethylase. It could be noticed that the 1,4-phenylene- bisthiazole moiety enters the depth of the catalytic site along the access channel, oblique to the active center of the enzyme, parallel to it at some distance. It is worth highlighting the difference in polarity of the binding regions from the access channel to the catalytic site of the enzyme. Two distinct areas of different polarity can be identified: a lipophilic, non-polar zone in the depth of the binding pocket, which contains the 1,4-phenylene-bisthiazole moiety, and a more polar one, located at the exit of the access channel to the active site of the enzyme, which contains the acylhydrazone group and the diverse substituted phenyl ring or thienyl moiety.

**Figure 2:**
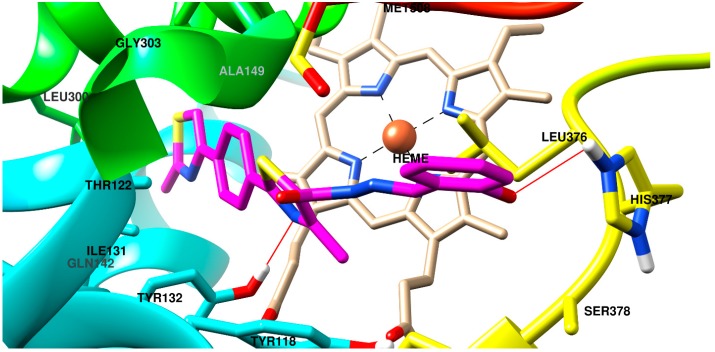
Docking pose of the compound **5d** in the binding site of lanosterol 14α-demethylase.

**Figure 3:**
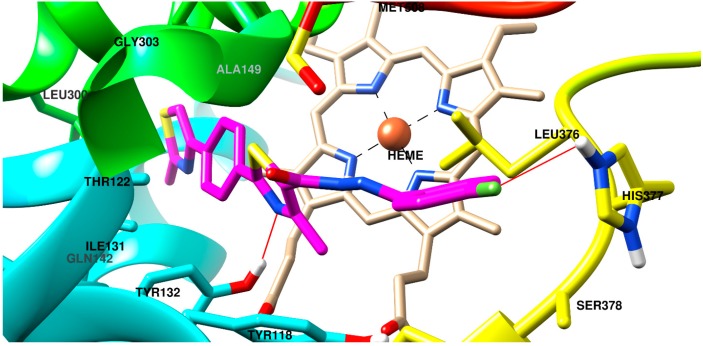
Docking pose of the compound **5f** in the binding site of lanosterol 14α-demethylase.

The polar area of the access channel contains polar amino acid residues (Tyr132, Tyr118, His377), with which the ligands can interact if the phenyl ring (Ar) is optimally substituted. Substitution of the nucleus with polar groups (*o*-OH, *p*-F) will lead to an optimal interaction between the molecules and His377 through a hydrogen bond, as shown in Figure 2 and Figure 3.

**Figure 4:**
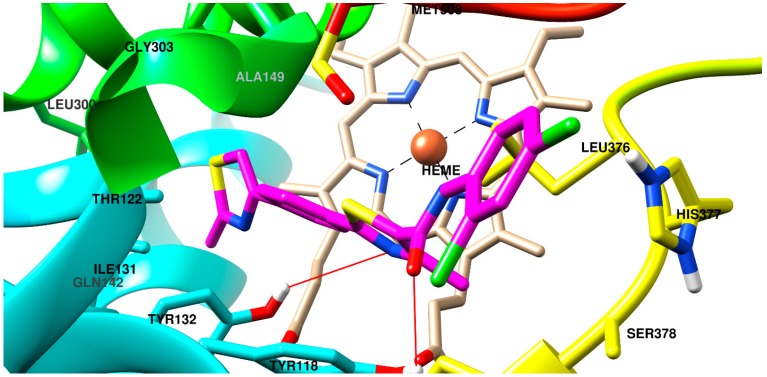
Docking pose of the compound **5h** in the binding site of lanosterol 14α-demethylase.

Substitution of the aryl group with non-polar or voluminous substituents will lead to repulsion due to His377 residue, and a mild but significant repositioning of the molecule in the binding pocket. For instance, compound **5h** is located nearer to the exit of the access channel, compared to **5d** or **5f**. Due to the particular positioning of the molecule and the consequent conformational changes in its structure, the occurrence of hydrogen bonds between Tyr118 and C=O from the acyl-hydrazone group could be observed as a novelty (Figure 4).

When the phenyl ring (Ar) is substituted with voluminous groups, the molecule gathers a long length and can no longer fit between the depth of the pocket and His377. Thus, different interactions could be observed between the compounds and the amino acid residues of the binding pocket, depending on how the phenyl ring is substituted, which could explain the differences in binding affinity of the tested compounds.

The newly synthesized compounds **5a**–**i** are predicted to bind to lanosterol 14α-demethylase in a different manner than clinically approved azoles, as long as there was no interaction between the tested compounds and the heme Fe^2+^ from the active site of the enzyme. All interactions which took place between the tested compounds and lanosterol 14α-demethylase involve amino acid residues from the access channel of the enzyme.

### 2.4. In Silico ADMET Evaluation

Drug-likeness of a molecule is a qualitative concept in designing new drugs, based on the theoretical calculation of certain parameters, giving a better assumption and understanding of the pharmacological activity of the compound [24]. Moreover, many drug candidates fail in late-phase studies due to an inadequate pharmacokinetic and pharmacotoxicological profile. Therefore, ADMET properties prediction has become an important approach in the drug discovery domain [25].

The drug-like properties of the synthesized compounds **5a**–**i**, according to the Lipinski Rule of Five, are summarized in Table 4. The predicted water solubility, gastrointestinal absorption, blood-brain barrier permeability, P-glycoprotein and CYP2D6 enzyme inhibition are presented in Table 5.

All newly synthesized acylhydrazone derivatives obey, without any violations, the Lipinski Rule of Five, which provides that, for a good absorption and permeation, compounds should have a MW less than 500 Da, no more than 5 HBDs, 10 HBAs and mLogP value under 4.15 [26]. The molar refractivity for all compounds is less than 130.

Topological polar surface area (TPSA) is a descriptor that is correlated with passive transport across the cell membranes [27]. Compounds **5a**, **5b**, **5c**, **5f**, **5g** and **5h**, that contain a phenyl ring substituted with a methoxy group or one or two halogen atoms, have TPSA values under 140 Å^2^, being associated with higher gastrointestinal absorption, compared to compounds **5d**, **5e** and **5i**, containing a phenyl ring substituted with a hydroxyl group or a thienyl residue, which have TPSA values above 140 Å^2^.

Percentage of gastrointestinal absorption (%Abs) varies from 56 to 66%, which indicates a moderate to good oral bioavailability. The ability of the compounds to pass the blood-brain barrier is absent, this being correlated with a reduced risk of developing side effects on the central nervous system, upon administration. Compounds are assumed to have a poor solubility in water, with logS values between −7.76 and −9.01.

P-glycoprotein (P-gp), a member of the ABC superfamily, plays an important role in intestinal absorption after oral administration, in biliary and renal excretion, as well as in avoiding brain penetration of drugs. Being a substrate to P-glycoprotein can generate a lot of drug-drug interactions and seriously modify the bioavailability and safety of a certain drug [28]. Our tested compounds did not seem to be substrates for P-gp.

Moreover, azole antifungal agents are known for their lack of specificity for the fungal enzyme. CYP51 is part of cytochrome P450 superfamily, found both in human and fungal cells. Therefore, it is important to know the possible pharmacokinetic interactions in which the newly synthesized compounds can be involved [29]. All tested compounds act as non-inhibitors of CYP2D6, so drug-drug interactions with compounds that are metabolized by this isoform of CYP450 superfamily are less likely to appear.

## 3. Materials and Methods

### 3.1. General Information

Reagents and solvents used in this study were of analytical grade purity and purchased from Sigma-Aldrich Chemicals (Buchs, Switzerland) and Alfa Aesar (Karlsruhe, Germany). The progress of all reactions was checked by thin layer chromatography (TLC) using precoated Silica Gel 60F_254_ sheets (Merck, Darmstadt, Germany). The spots on plates were visualized under UV light (254 nm). Melting points (m.p.) were determined using an MPM-H1 melting point apparatus (Schorpp Gerätetechnik, Überlingen, Germany) and are uncorrected. Elemental analyses for C, H, N and S were carried out on a Vario El CHNS instrument (Hanau, Germany). FT-IR spectra were recorded on a JASCO FT-IR-6100 spectrophotometer (Cremella, Italy). ^1^H-NMR and ^13^C-NMR analyses were performed on an Avance NMR spectrometer (Bruker, Karlsruhe, Germany), operating at 500 MHz and 125 MHz respectively, in DMSO-*d*_6_, using tetramethylsilane (TMS) as internal standard. Chemical shifts (δ values) are expressed in parts per million (ppm). M + 1 peaks were determined on an Agilent 1100 series and an Agilent Ion Trap SL mass spectrometer (Santa Clara, CA, USA), operating at 70 eV.

### 3.2. Chemistry

#### 3.2.1. Synthesis of 4-methyl-2-(4-(2-methylthiazol-4-yl)phenyl)thiazole-5-carbohydrazide (**4**)

To a solution of ethyl 4-methyl-2-(4-(2-methylthiazol-4-yl)phenyl)thiazole-5-carboxylate (**3**, 20 mmol, 6.88 g) in absolute ethanol (50 mL), hydrazine hydrate (40 mmol, 2.00 g) was added and the reaction mixture was refluxed on a water bath, until TLC indicated completion of the reaction (5 h). Then, the mixture was cooled down and the obtained precipitate was subjected to low pressure filtration, dried and recrystallized from ethanol. Yellow solid; 4.55 g; yield 69%; m.p. 224–226 °C; FT-IR (KBr) ν_max_ cm^−1^: 3291 (N–H str), 3102 (C–H thiazole str), 3038 (C–H str arom), 2921 (C–H str CH_3_), 1670 (C=O str), 1605 (C=N str); ^1^H-NMR (DMSO-*d*_6_) δ ppm: 9.61 (br, 1H, −NH−), 8.11 (s, 1H, thiazole-C_5_H), 8.08 (d, *J* = 8.4 Hz, 2H, phenyl), 7.99 (d, *J* = 8.4 Hz, 2H, phenyl), 4.63 (br, 2H, −NH_2_), 2.74 (s, 3H, −CH_3_), 2.61 (s, 3H, −CH_3_); ^13^C-NMR (DMSO-*d*_6_) δ ppm: 166.4 (C), 165.9 (C), 161.5 (C), 155.4 (C=O), 153.2 (C), 136.6 (C), 132.0 (C), 127.1 (2CH), 126.6 (C), 125.0 (2CH), 115.9 (CH), 19.4 (CH_3_), 17.4 (CH_3_); Anal. Calcd. for C_15_H_14_N_4_OS_2_ (%): C, 54.53; H, 4.27; N, 16.96; S, 19.41 Found (%): C, 54.48; H, 4.35; N, 16.79; S, 19.48; MS (ESI, 70 eV): *m*/*z* 331.1 (M + 1).

#### 3.2.2. General Procedure for the Synthesis of 1,4-phenylene-bisthiazole acyl-hydrazones **5a**–**i**

Equimolar quantities of carbohydrazide **4** and an appropriate aldehyde, in the presence of a catalytic amount of concentrated sulfuric acid (1 drop), were refluxed, in absolute ethanol (20 mL). When TLC indicated completion of the reaction (after 4 h), the mixture was cooled to room temperature. The resulting precipitate was filtered under vacuum and recrystallized from ethanol to obtain the pure compounds **5a**–**i**.

*N′-(2-Methoxybenzylidene)-4-methyl-2-(4-(2-methylthiazol-4-yl)phenyl)thiazole-5-carbohydrazide* (**5a**): Yellow solid; 0.353 g; yield 79%; m.p. 278–279 °C; FT-IR (KBr) ν_max_ cm^−1^: 3274 (N–H str), 3110 (C–H thiazole str), 3039 (C–H str arom), 2929 (C–H str CH_3_), 1682 (C=O str), 1605 (C=N str), 1250 (C–O–C assym str), 1025 (C–O–C sym str); ^1^H-NMR (DMSO-*d*_6_) δ ppm: 11.89 (br, 1H, −NH−), 8.26 (s, 1H, =CH−), 8.12–8.05 (m, 5H, thiazole-C_5_H, 4H-phenyl), 7.67 (d, *J* = 8.5 Hz, 1H, phenyl), 7.42 (m, 1H, phenyl), 7.18 (d, *J* = 8.1 Hz, 1H, phenyl), 7.12 (m, 1H, phenyl), 3.90 (s, 3H, −CH_3_), 2.79 (s, 3H, −CH_3_), 2.73 (s, 3H, −CH_3_); ^13^C-NMR (DMSO-*d*_6_) δ ppm: 167.0 (C), 166.4 (C), 162.4 (C), 161.9 (C), 161.0 (C=O), 153.4 (C), 143.8 (C=N), 136.8 (C), 134.3 (CH), 132.4 (C), 128.5 (CH), 127.2 (2CH), 126.3 (C), 125.2 (2CH), 122.8 (CH), 119.9 (C), 115.7 (CH), 113.7 (CH), 56.1 (CH_3_), 19.4 (CH_3_), 17.6 (CH_3_); Anal. Calcd. for C_23_H_20_N_4_O_2_S_2_ (%): C, 61.59; H, 4.49; N, 12.49; S, 14.29 Found (%): C, 61.74; H, 4.33; N, 12.52; S, 14.24; MS (ESI, 70 eV): *m*/*z* 449.3 (M + 1).

*N′-(3-Methoxybenzylidene)-4-methyl-2-(4-(2-methylthiazol-4-yl)phenyl)thiazole-5-carbohydrazide* (**5b**): Yellow solid; 0.376 g; yield 84%; m.p. 244 °C; FT-IR (KBr) ν_max_ cm^−1^: 3271 (N–H str), 3112 (C–H thiazole str), 3048 (C–H str arom), 2933 (C–H str CH_3_), 1675 (C=O str), 1598 (C=N str), 1265 (C–O–C assym str), 1039 (C–O–C sym str); ^1^H-NMR (DMSO-*d*_6_) δ ppm: 11.87 (br, 1H, −NH−), 8.18 (s, 1H, =CH−), 8.11–8.05 (m, 5H, thiazole-C_5_H, 4H-phenyl), 7.49 (d, *J* = 8.6 Hz, 1H, phenyl), 7.27 (m, 1H, phenyl), 7.18 (s, 1H, phenyl), 7.10 (d, *J* = 8.2 Hz, 1H, phenyl), 3.85 (s, 3H, −CH_3_), 2.79 (s, 3H, −CH_3_), 2.72 (s, 3H, −CH_3_); ^13^C-NMR (DMSO-*d*_6_) δ ppm: 169.2 (C), 166.4 (C), 162.5 (C), 162.0 (C), 161.2 (C=O), 153.3 (C), 144.7 (C=N), 136.6 (C), 134.0 (C), 132.3 (C), 129.2 (CH) 127.3 (2CH), 126.1 (C), 125.4 (2CH), 121.9 (CH), 117.2 (CH), 115.8 (CH), 111.3 (CH), 55.9 (CH_3_), 19.4 (CH_3_), 17.6 (CH_3_); Anal. Calcd. for C_23_H_20_N_4_O_2_S_2_ (%): C, 61.59; H, 4.49; N, 12.49; S, 14.29 Found (%): C, 61.67; H, 4.31; N, 12.37; S, 14.37; MS (ESI, 70 eV): *m*/*z* 449.1 (M + 1).

*N′-(4-Methoxybenzylidene)-4-methyl-2-(4-(2-methylthiazol-4-yl)phenyl)thiazole-5-carbohydrazide* (**5c**): Yellow solid; 0.358 g; yield 80%; m.p. 256–257 °C; FT-IR (KBr) ν_max_ cm^−1^: 3266 (N–H str), 3095 (C–H thiazole str), 3036 (C–H str arom), 2926 (C–H str CH_3_), 1658 (C=O str), 1606 (C=N str), 1252 (C–O–C assym str), 1034 (C–O–C sym str); ^1^H-NMR (DMSO-*d*_6_) δ ppm: 11.75 (br, 1H, −NH−), 8.13–8.08 (m, 6H, =CH−, thiazole-C_5_H, 4H-phenyl), 7.74 (d, *J* = 7.9 Hz, 2H, phenyl), 7.09 (d, *J* = 7.9 Hz, 2H, phenyl), 3.83 (s, 3H, −CH_3_), 2.79 (s, 3H, −CH_3_), 2.75 (s, 3H, −CH_3_); ^13^C-NMR (DMSO-*d*_6_) δ ppm: 170.0 (C), 166.4 (C), 162.3 (C), 161.7 (C), 161.2 (C=O), 153.3 (C), 144.1 (C=N), 136.7 (C), 132.5 (C), 129.3 (2CH), 127.3 (2CH), 126.8 (C), 126.2 (C), 125.5 (2CH), 115.8 (CH), 115.0 (2CH), 55.8 (CH_3_), 19.4 (CH_3_), 17.6 (CH_3_); Anal. Calcd. for C_23_H_20_N_4_O_2_S_2_ (%): C, 61.59; H, 4.49; N, 12.49; S, 14.29 Found (%): C, 61.72; H, 4.39; N, 12.54; S, 14.22; MS (ESI, 70 eV): *m*/*z* 449.2 (M + 1).

*N′-(2-Hydroxybenzylidene)-4-methyl-2-(4-(2-methylthiazol-4-yl)phenyl)thiazole-5-carbohydrazide* (**5d**): Yellow solid; 0.355 g; yield 82%; m.p. 266–267 °C; FT-IR (KBr) ν_max_ cm^−1^: 3419 (O–H str), 3277 (N–H str), 3104 (C–H thiazole str), 3048 (C–H str arom), 2939 (C–H str CH_3_), 1672 (C=O str), 1604 (C=N str); ^1^H-NMR (DMSO-*d*_6_) δ ppm: 11.90 (br, 1H, −NH−), 9.77 (br, 1H, OH), 8.28 (s, 1H, =CH−), 8.13–8.05 (m, 5H, thiazole-C_5_H, 4H-phenyl), 7.54 (d, *J* = 8.5 Hz, 1H, phenyl), 7.18 (m, 1H, phenyl), 7.08 (m, 1H, phenyl), 6.99 (d, *J* = 8.0 Hz, 1H, phenyl), 2.77 (s, 3H, −CH_3_), 2.72 (s, 3H, −CH_3_); ^13^C-NMR (DMSO-*d*_6_) δ ppm: 167.3 (C), 166.3 (C), 162.6 (C), 161.8 (C), 161.1 (C=O), 153.2 (C), 149.0 (C=N), 136.7 (C), 132.6 (C), 130.5 (CH), 129.4 (CH) 127.5 (2CH), 126.3 (C), 125.4 (2CH), 117.8 (CH), 116.6 (CH), 116.1 (C), 115.6 (CH), 19.4 (CH_3_), 17.5 (CH_3_); Anal. Calcd. for C_22_H_18_N_4_O_2_S_2_ (%): C, 60.81; H, 4.18; N, 12.89; S, 14.76 Found (%): C, 60.87; H, 4.06; N, 12.95; S, 14.87; MS (ESI, 70 eV): *m*/*z* 435.1 (M + 1).

*N′-(4-Hydroxybenzylidene)-4-methyl-2-(4-(2-methylthiazol-4-yl)phenyl)thiazole-5-carbohydrazide* (**5e**): Yellow solid; 0.368 g; yield 85%; m.p. 326–327 °C; FT-IR (KBr) ν_max_ cm^−1^: 3418 (O–H str), 3281 (N–H str), 3100 (C–H thiazole str), 3046 (C–H str arom), 2932 (C–H str CH_3_), 1660 (C=O str), 1608 (C=N str); ^1^H-NMR (DMSO-*d*_6_) δ ppm: 11.84 (br, 1H, −NH−), 9.75 (br, 1H, OH), 8.12–8.05 (m, 6H, =CH−, thiazole-C_5_H, 4H-phenyl), 7.78 (d, *J* = 8.3 Hz, 2H, phenyl), 6.92 (d, *J* = 8.3 Hz, 2H, phenyl), 2.77 (s, 3H, −CH_3_), 2.72 (s, 3H, −CH_3_); ^13^C-NMR (DMSO-*d*_6_) δ ppm: 168.0 (C), 166.3 (C), 162.5 (C), 161.9 (C), 161.3 (C=O), 153.1 (C), 144.9 (C=N), 136.8 (C), 132.7 (C), 131.0 (2CH), 127.5 (2CH), 127.0 (C), 126.3 (C), 125.4 (2CH), 116.9 (2CH), 115.6 (CH), 19.4 (CH_3_), 17.5 (CH_3_); Anal. Calcd. for C_22_H_18_N_4_O_2_S_2_ (%): C, 60.81; H, 4.18; N, 12.89; S, 14.76 Found (%): C, 60.68; H, 4.11; N, 12.97; S, 14.88; MS (ESI, 70 eV): *m*/*z* 435.2 (M + 1).

*N′-(4-Fluorobenzylidene)-4-methyl-2-(4-(2-methylthiazol-4-yl)phenyl)thiazole-5-carbohydrazide* (**5f**): Yellow solid; 0.388 g; yield 89%; m.p. 316 °C; FT-IR (KBr) ν_max_ cm^−1^: 3264 (N–H str), 3103 (C–H thiazole str), 3038 (C–H str arom), 2923 (C–H str CH_3_), 1663 (C=O str), 1611 (C=N str), 1231 (C–F); ^1^H-NMR (DMSO-*d*_6_) δ ppm: 11.87 (br, 1H, −NH−), 8.13–8.06 (m, 6H, =CH−, thiazole-C_5_H, 4H-phenyl), 7.72 (d, *J* = 8.1 Hz, 2H, phenyl), 7.08 (d, *J* = 8.1 Hz, 2H, phenyl), 2.79 (s, 3H, −CH_3_), 2.71 (s, 3H, −CH_3_); ^13^C-NMR (DMSO-*d*_6_) δ ppm: 169.1 (C), 166.5 (C), 162.5 (C), 161.6 (C), 160.7 (C=O), 153.6 (C), 143.4 (C=N), 136.7 (C), 132.6 (C), 131.2 (2CH), 128.4 (C), 127.3 (2CH), 126.4 (C), 125.6 (2CH), 115.5 (CH), 115.0 (2CH), 19.4 (CH_3_), 17.6 (CH_3_); Anal. Calcd. for C_22_H_17_FN_4_OS_2_ (%): C, 60.53; H, 3.93; N, 12.84; S, 14.69 Found (%): C, 60.66; H, 3.89; N, 12.75; S, 14.74; MS (ESI, 70 eV): *m*/*z* 437.2 (M + 1).

*N′-(4-Chlorobenzylidene)-4-methyl-2-(4-(2-methylthiazol-4-yl)phenyl)thiazole-5-carbohydrazide* (**5g**): Yellow solid; 0.329 g; yield 73%; m.p. 295 °C; FT-IR (KBr) ν_max_ cm^−1^: 3264 (N–H str), 3101 (C–H thiazole str), 3046 (C–H str arom), 2935 (C–H str CH_3_), 1678 (C=O str), 1603 (C=N str), 743 (C–Cl); ^1^H-NMR (DMSO-*d*_6_) δ ppm: 11.79 (br, 1H, −NH−), 8.12–8.06 (m, 6H, =CH−, thiazole-C_5_H, 4H-phenyl), 7.79 (d, *J* = 8.4 Hz, 2H, phenyl), 7.06 (d, *J* = 8.4 Hz, 2H, phenyl), 2.78 (s, 3H, −CH_3_), 2.73 (s, 3H, −CH_3_); ^13^C-NMR (DMSO-*d*_6_) δ ppm: 166.4 (C), 162.6 (C), 162.1 (C), 161.0 (C=O), 153.4 (C), 142.6 (C=N), 136.5 (C), 132.5 (C), 131.7 (C), 131.1 (2CH), 130.1 (2CH), 129.6 (C), 127.4 (2CH), 126.2 (C), 125.5 (2CH), 115.7 (CH), 19.4 (CH_3_), 17.5 (CH_3_); Anal. Calcd. for C_22_H_17_ClN_4_OS_2_ (%): C, 58.33; H, 3.78; N, 12.37; S, 14.16 Found (%): C, 58.51; H, 3.68; N, 12.19; S, 14.25; MS (ESI, 70 eV): *m*/*z* 453.4 (M + 1).

*N′-(2,4-Dichlorobenzylidene)-4-methyl-2-(4-(2-methylthiazol-4-yl)phenyl)thiazole-5-carbohydrazide* (**5h**): Yellow solid; 0.384 g; yield 79%; m.p. 331–332 °C; FT-IR (KBr) ν_max_ cm^−1^: 3241 (N–H str), 3094 (C–H thiazole str), 3050 (C–H str arom), 2945 (C–H str CH_3_), 1667 (C=O str), 1607 (C=N str), 739, 701 (2C–Cl); ^1^H-NMR (DMSO-*d*_6_) δ ppm: 11.93 (br, 1H, −NH−), 8.38 (s, 1H, =CH−), 8.14–8.07 (m, 5H, thiazole-C_5_H, 4H-phenyl), 7.92 (d, *J* = 8.6 Hz, 1H, phenyl), 7.65 (s, 1H, phenyl), 7.38 (d, *J* = 8.6 Hz, 1H, phenyl), 2.80 (s, 3H, −CH_3_), 2.74 (s, 3H, −CH_3_); ^13^C-NMR (DMSO-*d*_6_) δ ppm: 166.5 (C), 162.7 (C), 162.0 (C), 161.1 (C=O), 153.4 (C), 141.3 (C=N), 136.6 (C), 132.6 (C), 132.0 (C), 131.9 (C), 131.0 (CH), 130.1 (CH), 128.1 (C), 127.5 (2CH), 126.8 (C), 126.1 (CH), 125.5 (2CH), 115.7 (CH), 19.4 (CH_3_), 17.5 (CH_3_); Anal. Calcd. for C_22_H_16_Cl_2_N_4_OS_2_ (%): C, 54.21; H, 3.31; N, 11.49; S, 13.16 Found (%): C, 54.18; H, 3.39; N, 11.53; S, 13.11; MS (ESI, 70 eV): *m*/*z* 487.2 (M + 1).

*4-Methyl-2-(4-(2-methylthiazol-4-yl)phenyl)-N′-(thiophen-2-ylmethylene)thiazole-5-carbohydrazide* (**5i**): Yellow solid; 0.377 g; yield 89%; m.p. 292 °C; FT-IR (KBr) ν_max_ cm^−1^: 3274 (N–H str), 3108 (C–H thiazole str), 3034 (C–H str arom), 2921 (C–H str CH_3_), 1674 (C=O str), 1607 (C=N str); ^1^H-NMR (DMSO-*d*_6_) δ ppm: 11.86 (br, 1H, −NH−), 8.31 (br, 1H, =CH−), 8.13–8.06 (m, 5H, thiazole-C_5_H, 4H-phenyl), 7.72 (d, *J* = 8.1 Hz, 1H, thienyl), 7.50 (d, *J* = 8.6 Hz, 1H, thienyl), 7.17 (m, 1H, thienyl), 2.78 (s, 3H, −CH_3_), 2.72 (s, 3H, −CH_3_); ^13^C-NMR (DMSO-*d*_6_) δ ppm: 166.4 (C), 162.3 (C), 161.8 (C), 161.3 (C=O), 153.2 (C), 139.3 (C=N), 136.8 (C), 136.3 (C), 132.4 (C), 129.7 (CH), 128.5 (CH), 127.5 (2CH), 127.0 (CH), 126.0 (C), 124.7 (2CH), 115.9 (CH), 19.4 (CH_3_), 17.5 (CH_3_); Anal. Calcd. for C_20_H_16_N_4_OS_3_ (%): C, 56.58; H, 3.80; N, 13.20; S, 22.65 Found (%): C, 56.42; H, 3.69; N, 13.36; S, 22.77; MS (ESI, 70 eV): *m*/*z* 425.2 (M + 1).

### 3.3. Anti-Candida Activity Assay

Evaluation of the anti-*Candida* activity was done according to the guidelines of Clinical Laboratory Standards Institute (CLSI) [30]. In order to determine the minimum inhibitory concentration (MIC) and minimum fungicidal concentration (MFC) values of the tested compounds, *Candida albicans* ATCC 10231, *Candida albicans* ATCC 18804, *Candida krusei* ATCC 6258 and *Candida parapsilosis* ATCC 22019 were used. All the tested fungal strains were obtained from Food Biotechnology Laboratory, Life Sciences Institute, University of Agricultural Sciences and Veterinary Medicine Cluj-Napoca, Romania. The cultures were stored on potato dextrose agar (Sifin, Germany). Prior to antifungal susceptibility testing, each strain was inoculated on potato dextrose agar plates to ensure optical growth characteristics and purity. The medium used for susceptibility testing was Roswell Park Memorial Institute (RPMI) 1640 with l-glutamine, adjusted to pH 7.0 with 3-(*n*-morpholino)propanesulfonic acid. The initial density of *Candida* spp. was approximately 2 × 10^6^ colony forming units/mL (CFU/mL). Inoculums (density of 0.5 in McFarland scale) were prepared in a 0.9% NaCl sterile solution. Then, tested strains were suspended in RPMI 1640 medium, to give a final density of 2 × 10^3^ CFU/mL.

Stock solutions (1 mg/mL) were obtained by dissolving the newly synthesized compounds and the reference antifungal, fluconazole, in sterile DMSO. These solutions were stored at 4 °C. A series of double diluting solutions of the above compounds were prepared in RPMI 1640 medium obtaining final concentrations in the range of 500–0.015 µg/mL. The broth microdilution method was employed for the minimum inhibitory concentration test. The medium was placed into each of the 96 wells of the microplates. Sample solutions at high concentration were added into the first rows of the microplates and two-fold dilutions of the compounds were made by dispensing the solutions into the remaining wells. 10 µL of the culture suspensions were inoculated into the wells. The growth control, sterility control and control of antifungal compound were used. Plates were incubated under normal atmospheric conditions at 30 °C for 48 h. Minimum inhibitory concentration (MIC) values have been determined by adding resazurin (20 μL, 0.02%) followed by incubation for 2 h. The MIC was defined as the lowest concentration required arresting the growth of the fungal strain. For determination of minimum fungicidal concentration (MFC), 0.01 mL of the medium withdrawn from the culture tubes, showing no macroscopic growth at the end of the 24 h, was subcultured on potato dextrose agar plates to determine the number of vital organisms and incubated further at 30 °C for 48 h. The MFC was defined as the lowest concentration of the agent at which no colonies are observed.

### 3.4. Molecular Docking

The molecular docking study targeted the compounds **5a**–**i** into the active site of fungal lanosterol 14α-demethylase, using AutoDock 4.2.6 [31], in order to evaluate their binding affinity for the protein and to predict the binding poses of the new acylhydrazone derivatives. Furthermore, focusing on understanding the differences between compounds in terms of substituents’ variability and how different groups might influence the protein-ligand interactions, we decided to keep the same target protein and docking protocol as reported in our previous work [17]. The macromolecular target was built by homology modeling using the three-dimensional structure 5EQB from Protein Data Bank (www.rcsb.org). The search space size (x, y, z) was set to 75 × 75 × 75 points with a 0.375 Å grid spacing, with the center Cartesian coordinates set to x = −23.134, y = 13.943, z = 19.959.

The unique change in our protocol was the increasing of the generated conformations to 100 for each ligand, in order to increase the precision and to obtain more realistic results. The inhibition constant (Ki) was calculated using the formula $K_{i}=e^{\frac{\Delta G\times 1000}{R\times T}}$, where Δ*G* represents the computed binding affinity energy, *R* = 1.98719 cal mol^−1^ K^−1^ (Regnault constant) and *T* = 298.15 *K* = 25 °C. Clustering analysis of the resulted conformations was performed in order to analyze and to evaluate the homogeneity of binding of the ligands to the catalytic site of the enzyme [17,32]. Visualization of the docking results was performed using UCSF Chimera [33].

### 3.5. In Silico ADMET Evaluation

Theoretical in silico evaluation of ADMET properties of compounds **5a**–**i** was performed using the Swiss-ADME online platform [34,35]. In this study, we assessed the following descriptors, taking into consideration the Lipinski Rule of Five: molecular weight (MW), number of rotatable bonds (RoB), hydrogen bond acceptors (HBA) and hydrogen bond donors (HBD), Moriguchi LogP (mLogP) and molar refractivity [26]. Water solubility, topological polar surface area and gastrointestinal absorption, which are predictors of the oral bioavailability of a certain compound, the capacity to inhibit CYP2D6 and P-glycoprotein, which are involved in many drug-drug interactions, and the ability of the compounds to cross the blood-brain barrier were also investigated [27]. Percentage of gastrointestinal absorption (%Abs) was calculated using the formula: %Abs = 109 − 0.345 × TPSA [36].

## 4. Conclusions

Nine new acylhydrazone derivatives **5a**–**i** were designed, synthesized, fully characterized and evaluated for their inhibitory activity against different *Candida* spp. In the in vitro anti-*Candida* activity assay performed, compounds **5d** and **5f** exhibited the best inhibitory activity against both *C. albicans* strains and compound **5g** displayed the highest inhibition against *C. parapsilosis*. The most susceptible fungal strain to the newly synthesized compounds was *C. krusei*, four out of nine compounds being as active as the reference drug used, fluconazole, against this *Candida* strain.

A molecular docking study was performed in order to investigate the binding poses of the new compounds **5a**–**i** towards lanosterol 14α-demethylase. The obtained results from the docking simulations showed that our compounds interact with the amino acid residues from the access channel to the active site of the enzyme, with no interaction with amino acids from the catalytic site.

Analyzing the results of the in silico ADMET screening, it can be observed that the new synthesized acylhydrazone derivatives displayed favorable drug-like properties according to Lipinski Rule of Five, making them suitable for development as oral drug candidates. Moreover, the compounds are not predicted to pass the blood-brain barrier or to inhibit CYP2D6 and P-glycoprotein, so the risk for central nervous system side effects and drug-drug interactions is considerably low. All in all, the above-mentioned observations provide a promising basis for the development of new anti-*Candida* agents.

## Figures and Tables

**Table 1:** The minimum inhibitory concentration values (MIC, µg/mL) of the newly synthesized compounds **5a**–**i** against all tested *Candida* strains.

Compound	*C. albicans* ATCC 10231	*C. albicans* ATCC 18804	*C. krusei* ATCC 6258	*C. parapsilosis* ATCC 22019
**5a**	31.25	31.25	62.5	62.5
**5b**	31.25	31.25	62.5	62.5
**5c**	31.25	31.25	31.25	31.25
**5d**	**15.62**	**15.62**	**15.62**	15.62
**5e**	31.25	31.25	31.25	31.25
**5f**	**15.62**	**15.62**	**15.62**	15.62
**5g**	31.25	31.25	**15.62**	**7.81**
**5h**	31.25	31.25	**15.62**	15.62
**5i**	31.25	31.25	31.25	31.25
Fluconazole	15.62	15.62	15.62	7.81
Drug-free	Growth in all concentrations			
Broth control	No growth			

The results obtained for the most active compounds are marked in bold.

**Table 2:** The minimum fungicidal concentration values (MFC, µg/mL) of the newly synthesized compounds **5a**–**i** against all tested *Candida* strains.

Compound	*C. albicans* ATCC 10231	*C. albicans* ATCC 18804	*C. krusei* ATCC 6258	*C. parapsilosis* ATCC 22019
**5a**	62.5	62.5	125	125
**5b**	62.5	62.5	125	125
**5c**	62.5	62.5	62.5	62.5
**5d**	**31.25**	**31.25**	**31.25**	31.25
**5e**	62.5	62.5	62.5	62.5
**5f**	**31.25**	**31.25**	**31.25**	31.25
**5g**	62.5	62.5	**31.25**	**15.62**
**5h**	62.5	62.5	**31.25**	31.25
**5i**	62.5	62.5	62.5	62.5
Fluconazole	31.25	31.25	31.25	15.62
Drug-free	Growth in all concentrations			
Broth control	No growth			

The results obtained for the most active compounds are marked in bold.

**Table 3:** Predicted binding affinity (ΔG), inhibition constant (Ki) and cluster analysis of the tested compounds **5a**–**i**.

Compound	Best Binding Conformation		2Å Cluster of the Best Binding Conformation				Number of Other Clusters
	ΔG (kcal/mol)	Ki (nM)	NoC ^1^	Average ΔG (kcal/mol)	Standard Deviation		
					ΔG (kcal/mol)	RMSD ^2^ of Cartesian Coordinates	
**5a**	−11.86	2.03	74	−11.68	0.39	0.44	8
**5b**	−11.92	1.83	66	−11.82	0.38	0.43	5
**5c**	−11.87	1.99	65	−11.66	0.24	0.56	6
**5d**	−12.16	1.22	81	−11.91	0.30	0.39	4
**5e**	−11.97	1.68	70	−11.89	0.30	0.48	7
**5f**	−11.46	3.98	77	−11.29	0.14	0.25	3
**5g**	−11.98	1.65	68	−11.82	0.32	0.48	3
**5h**	−12.36	0.87	76	−12.17	0.26	0.31	6
**5i**	−11.85	2.06	65	−11.52	0.36	0.45	8

^1^ NoC = number of conformations; ^2^ RMSD = root-mean-square deviation.

**Table 4:** In silico drug-like properties of compounds **5a**–**i**, according to Lipinski Rule of Five.

Compound	MW ^1^ (Da)	Rob ^2^	HBA ^3^	HBD ^4^	MR ^5^	TPSA ^6^	mLogP ^7^	Lipinski Violations
**5a**	448.56	7	5	1	126.25	132.95	2.25	0
**5b**	448.56	7	5	1	126.25	132.95	2.25	0
**5c**	448.56	7	5	1	126.25	132.95	2.25	0
**5d**	434.53	6	5	2	121.78	143.95	2.04	0
**5e**	434.53	6	5	2	121.78	143.95	2.04	0
**5f**	436.52	6	5	1	119.72	123.72	2.96	0
**5g**	452.98	6	4	1	124.77	123.72	3.06	0
**5h**	487.42	6	4	1	129.78	123.72	3.54	0
**5i**	424.56	6	4	1	117.64	151.96	2.17	0

Abbreviations: ^1^ Molecular weight; ^2^ Number of rotatable bonds; ^3^ Number of hydrogen bond acceptors; ^4^ Number of hydrogen bond donors; ^5^ Molar refractivity; ^6^ Topological polar surface area; ^7^ Moriguchi logarithm of the compound partition coefficient between *n*-octanol and water.

**Table 5:** In silico pharmacokinetic properties of compounds **5a**–**i**.

Compound	logS ^1^	%Abs ^2^	BBBP ^3^	Pgp ^4^ Substrate	CYP2D6 Inhibitor
**5a**	−7.86	63.13	No	No	No
**5b**	−7.86	63.13	No	No	No
**5c**	−7.86	63.13	No	No	No
**5d**	−7.76	59.33	No	No	No
**5e**	−7.76	59.33	No	No	No
**5f**	−7.8	66.31	No	No	No
**5g**	−8.35	66.31	No	No	No
**5h**	−9.01	66.31	No	No	No
**5i**	−8.31	56.57	No	No	No

Abbreviations: ^1^ logarithm of water solubility; ^2^ gastrointestinal absorption; ^3^ blood-brain barrier permeability; ^4^ P-glycoprotein.
